# Emergency Military Hospital Construction

**Published:** 1915-11-20

**Authors:** 


### Emergency Military Hospital Construction.

Mr. A. Saxon Snell, F.R.I.B.A., was down to give a lecture under this title, illustrated by lantern slides, at the Royal Society of Medicine on the afternoon of the 10th instant. There was a large attendance, which consisted principally of orderlies and nurses, with a few R.A.M.C. doctors and several architects. Mr. Snell did not, in fact, deliver a lecture, but confined himself mainly to lantern slides relating to existing temporary war hospitals, with a number of block plans and some sections of existing wards. He mentioned especially the 1st Eastern Hospital at Cambridge, and the 5th Northern General Hospital at Leicester. Mr. Snell summarised "the buildings of war hospitals which are meant to be temporary, built quickly, and at very small cost," features which are "incompatible with refinement in detail." He made a good point by insisting that a layer of concrete for the whole surface of the ground on which temporary buildings are erected is essential. This is so, for even when a building is raised a few feet above the ground, providing a clear sweep for the air, especially in the case of enclosed wards, "ground air is apt to find its way upwards, and the quality of ground air is variable and not to be ignored with impunity." Mr. John Slater, F.R.I.B.A., took the chair, and in thanking Mr. Snell for his lantern slides and for the plans exhibited on the walls, remarked that it would have been an advantage to some present if the plans exhibited had been left longer on the screen.

